# Practicing a Musical Instrument in Childhood is Associated with Enhanced Verbal Ability and Nonverbal Reasoning

**DOI:** 10.1371/journal.pone.0003566

**Published:** 2008-10-29

**Authors:** Marie Forgeard, Ellen Winner, Andrea Norton, Gottfried Schlaug

**Affiliations:** 1 Department of Neurology, Beth Israel Deaconess Medical Center and Harvard Medical School, Boston, Massachusetts, United States of America; 2 Department of Psychology, Boston College, Chestnut Hill, Massachusetts, United States of America; 3 Project Zero, Harvard Graduate School of Education, Cambridge, Massachusetts, United States of America; University of St. Andrews, United Kingdom

## Abstract

**Background:**

In this study we investigated the association between instrumental music training in childhood and outcomes closely related to music training as well as those more distantly related.

**Methodology/Principal Findings:**

Children who received at least three years (*M* = 4.6 years) of instrumental music training outperformed their control counterparts on two outcomes closely related to music (auditory discrimination abilities and fine motor skills) and on two outcomes distantly related to music (vocabulary and nonverbal reasoning skills). Duration of training also predicted these outcomes. Contrary to previous research, instrumental music training was not associated with heightened spatial skills, phonemic awareness, or mathematical abilities.

**Conclusions/Significance:**

While these results are correlational only, the strong predictive effect of training duration suggests that instrumental music training may enhance auditory discrimination, fine motor skills, vocabulary, and nonverbal reasoning. Alternative explanations for these results are discussed.

## Introduction

There is a widespread view that learning to play a musical instrument in childhood stimulates cognitive development and leads to enhanced skills in a wide variety of areas [1], [2]. The slogan that “music makes you smarter” has been fostered by reports from the College Board that scores on the SAT (a test of verbal and mathematical abilities required by most U.S. colleges) rise incrementally for each year of high school music instruction [3]. However, the results of other experimental and correlational studies investigating these claims have been conflicting.

The effect that training (or skill acquisition) in one domain might have on skills and cognitive performances in other domains is commonly referred to as transfer. The study of transfer has a long and contentious history [4]–[7]. The most commonly observed form of transfer occurs when there is a close resemblance between the training domain and the transfer domain (typically called “near transfer”) (e.g., learning to estimate the area of a square and understanding how to estimate the area of a triangle; learning to play a musical instrument and developing fine motor skills as well as melodic/rhythmic discrimination skills).

Although only experimental/longitudinal studies can demonstrate transfer, the results of many correlational studies have been used to suggest that transfer may occur from music training to other domains. We differentiate here between experimental, longitudinal studies and correlational studies testing for transfer.

Past research has clearly demonstrated that near transfer occurs from music training to music perception skills. In a longitudinal study, Flohr [8] showed that five year-olds who received twelve weeks of music instruction improved significantly more than control children in tonal and rhythmic auditory discrimination abilities. Furthermore, in a correlational study, Morrongiello and Roes [9] demonstrated that musically trained 9-year-olds were better at drawing melodic contours than untrained children.

There is also evidence for near transfer from instrumental music training to motor skills. A longitudinal investigation by Costa-Giomi [10] showed that children who received two years of piano instruction improved significantly more than controls on a motor proficiency test. In another longitudinal study, Hurwitz, Wolff, Bortnick, and Kokas [11] showed that after seven months of Kodály music instruction, children improved significantly more than a matched control group on a motor sequencing task in which they tapped keys synchronously with a metronome, and then continued to tap “in time” after the metronome was turned off. A correlational study by Jäncke, Schlaug, and Steinmetz [12] also showed that finger-tapping rates are faster in adult musicians than non-musicians, and the tapping rate of the non-dominant hand increases with duration of music training.

While near transfer effects are relatively common, it is notoriously difficult to demonstrate far transfer [4], [5], where the resemblance between training and transfer domains is much less obvious (e.g., learning to read musical rhythm notation and understanding fractions). Evidence for far transfer from instrumental music training has previously been reported in the areas of spatial, verbal, and mathematical performances, as well as general IQ, as described below. Although much of the evidence is correlational, a few studies have demonstrated far transfer experimentally.

### Music and Spatial Skills

A number of studies have investigated the relationship between music training and spatial abilities. Some have argued that spatial reasoning could be enhanced by music training because music notation itself is spatial, since specific pitches are indicated by their particular position on a series of lines and spaces [13], [14]. Others have argued that the proximity of brain regions for music and spatial processing may be responsible for transfer effects [15], [16]. A meta-analysis of 15 experimental studies by Hetland showed that music instruction enhances performance on certain spatial tasks (such as the Object Assembly subtest of the WISC) but not on Raven's Standard Progressive Matrices, a test of nonverbal reasoning with some visual-spatial elements [13]. Hetland also reported the results of correlational studies testing the association between music training and spatial outcomes: out of 13 studies, five reported a positive association between music training and spatial outcomes and eight had negative, null, or mixed results. The evidence for transfer effects from music training to spatial skills remains therefore mixed.

### Music and Verbal Skills

Parallels between music and language have been used to support the hypothesis that music training may strengthen verbal skills. Both music and written language involve formal notation read from left to right; music notation consists of symbols that represent information about sound (pitch, harmony, melody) and time (rhythm, meter), and listening to both music and speech requires attention to the temporal order of rapidly changing acoustic events [17], [18].

A number of correlational studies have reported an association between musical and language skills. Anvari, Trainor, Woodside and Levy [19] found that pitch perception was related to phonemic awareness and reading ability in five year-old children. Other researchers [20] found, after intellectual ability was controlled, that young adults with music training scored higher than those without music training on recall of both unfamiliar spoken and sung lyrics. The same researchers [21] also reported a positive correlation between years of music training and verbal recall of stories, as well as between years of music training and performance on auditory temporal order tests requiring discrimination of the order of tones and syllables. Auditory temporal order scores were found to mediate the relation between music training and verbal recall. Finally, in a meta-analysis of 25 correlational studies, Butzlaff [22] found a significant association between music training and reading skills.

The existence of a transfer effect between music training and language skills has also been supported by experimental studies, although the evidence is not unequivocal. In a study by Overy [23], [24], only phonological awareness (but not reading) was shown to improve in children with dyslexia after an intervention based on singing and rhythm games. Another group of researchers found that musically trained adult women [25] and musically trained children [26] outperformed those without music training on a verbal memory test (but not on a visual memory test). After one year, children who continued music training showed greater improvement in verbal memory while those who had discontinued training did not improve. However, the musically trained group had higher IQ scores (*p* = .09) as well as almost one more year of education (*p* = .12). In addition, since the words to be remembered were presented orally, it is possible that this memory effect is limited to auditory verbal memory. Finally, in addition to the meta-analysis of correlational studies described above, Butzlaff [22] conducted an additional meta-analysis based on six experimental studies (only two of which were published) testing whether music training improves reading ability. This second meta-analysis yielded a small significant effect but one that was not robust enough to show a causal relationship between music training and reading ability. As with spatial skills, the evidence supporting the existence of transfer effects from music training to language skills remains mixed.

### Music and Mathematical Skills

Although many explanations could be given for potential transfer between music training and mathematical performance (e.g., musical rhythm is based on mathematical relations), little experimental evidence has shown that such transfer occurs. A meta-analysis of six experimental studies testing whether music training leads to improved mathematics performance yielded a small, but significant overall effect size (*r* = .13) [3]. However, given that only two of the six studies showed a significant positive effect, Vaughn concluded that the hypothesis that music training enhances math performance has not yet been adequately put to the test. Gardiner, Fox, Knowles, and Jeffrey [27], whose study was not included in the aforementioned meta-analysis because it did not disentangle a music from a visual arts intervention, showed that first-graders who received both visual arts and music training over the course of seven months improved on mathematical outcomes and surpassed their control counterparts. However, this study could neither rule out the possibility that the children in the arts group had more effective teachers, nor, as mentioned, disentangle the effects of visual arts and music instruction. As with other cognitive domains, the evidence supporting the existence of transfer effects between music training and mathematical abilities remains mixed.

### Music and General IQ

Schellenberg [28] reported a positive correlation between music lessons and IQ in 6–11 year olds, and showed that taking music lessons as a child predicts both academic performance and IQ in young adulthood (holding constant family income and parents' education). In an experimental study, Schellenberg [29] also showed that a group of six year-olds who received keyboard or singing lessons in small groups for 36 weeks had significantly larger (although modest) increases in full-scale IQ and standardized educational achievement than did matched groups of children receiving either drama lessons or no lessons. Schellenberg argued that music lessons function as additional schooling—requiring focused attention, memorization, and the progressive mastery of technical skill. And it is well established that schooling increases IQ [30]. However, contrary to previous research, the group that showed the largest enhancement of IQ was the singing group, not the keyboard group. The evidence supporting the existence of transfer effects from instrumental music training to general IQ is therefore not yet clear.

Most of the studies investigating the effect of instrumental music training on cognitive abilities have not tested for near transfer. We argue here that any study of far transfer must include measures of near transfer in order to ascertain that learning in the parent domain has actually occurred. In addition, as shown above, the findings on far transfer are not always consistent, and hence further research is called for.

Here we report the results of a correlational study testing the hypotheses that children receiving instrumental music training perform better than those without such training in four areas of cognition that are distantly related to music: spatial, verbal, nonverbal, and mathematical. We also investigated whether children with music training perform better on two areas closely associated with music: fine motor and auditory skill. We also examined whether duration of training predicted performance on either the distant or closely associated tests. Finally, we discuss possible explanations for our findings and suggest which variables should be further investigated in future research.

## Methods

### Participants

Fifty-nine children were recruited from public schools and community music schools in the Boston area, as well as by word-of-mouth, to participate in this study which was approved by the Institutional Review Board (IRB) of Beth Israel Deaconess Medical Center (BIDMC). All children as well as their parents gave written informed consent to participate in this study. The mean age at the median testing session for each child was 9.96 years (*SD* = 0.74, range = 8.73 to 11.31 years). Forty-one children (19 boys, 22 girls, mean age = 10.10 years old, *SD* = 0.76, range = 8.73 to 11.31 years) who had completed a minimum of three years of instrumental music training formed the Instrumental group. The mean number of years training was 4.63 (*SD* = 1.10; range = 3.16 to 7.07 years). Twenty-two children played keyboard instruments, 12 children played string instruments (10 violin, 2 cello, and 1 double bass), and 6 children played both kinds of instruments (3 studied both piano and violin, 2 studied both piano and viola, and 1 studied both piano and cello). The Instrumental group was subdivided into two subgroups according to the type of instrumental instruction received. Twenty-one children in the Instrumental group received traditional instrumental instruction (in which children are taught to read music notation from the very beginning). The remaining 20 children in the Instrumental group received Suzuki instruction (in which playing by ear is first emphasized, and music notation is only introduced later in the curriculum). Eighteen children (7 girls, 11 boys, mean age = 9.63 years old, *SD* = 0.57, range = 9.08 to 10.96 years) who had received no instrumental music instruction formed the Control group. Children in both groups were exposed to general music classes in school, typically lasting for 30–40 minutes a week, but these classes included neither instrumental training nor one-on-one music instruction.

### Materials and Procedure

Children participated in 3–4 testing sessions (about six hours), over the course of 3–4 weeks.

#### Socio-Economic Status (SES)

Parents reported their highest level of education on a questionnaire and responses were scored on a 6-point scale: (1) some high school; (2) high school diploma or GED; (3) some college, vocational degree, or associate's degree; (4) 4-year college degree (BA, BS); (5) master's degree (MA, MS, MBA); (6) doctoral degree (PhD, MD, JD, EdD, ThD). Final SES scores represent either the single parent's score in a one-parent family, or the average of both parents' scores in a two-parent family. While education alone is not a complete measure of SES, it is considered to be an acceptable indicator [31].

#### Duration of Training

Duration of training for children in the Instrumental group (in weeks) was calculated from the child's first music lesson to the median time-point of the child's testing cycle.

#### Practice Intensity

In addition to reporting the date of commencement of music training, parents also indicated how much their children played their instrument(s) (in minutes per week), including home practice time as well as instruction and ensemble time. We specifically asked parents to estimate practice intensity a*t the time of testing* because we were aware that retrospective estimates might not be accurate.

#### Handedness

Handedness was assessed using four measures adapted from Annett [32], [33]. Hand dominance was determined by the use of the same hand for at least three of the four tasks. Those who completed two tasks with one hand and two with the other were classified as mixed-handers. The Control group was composed of only right-handers while the Instrumental group also included four left-handed boys and one left-handed girl.

#### Gordon's Intermediate Measures of Music Audiation (IMMA)

Children received Gordon's Intermediate Measures of Music Audiation (IMMA) [34], which consists of 40 pairs of tone sequences and 40 pairs of rhythms. Children make a same/different judgment by circling a pair of same or different faces on the answer sheet.

#### Melodic and Rhythmic Discrimination task

A Melodic/Rhythmic Discrimination task designed in our lab [33], [35] was also administered. Children indicated whether two melodic or rhythmic phrases of 5 tones each were the same or different. In contrast to the sine-wave tones used in Gordon's IMMA, our own stimuli used the sound of an actual musical instrument (marimba) so that both attack and release could be discerned. Unlike the Gordon's stimuli, which varied in overall length, our stimuli were identical in overall duration. This served to clarify the metric context so that children could focus on melodic or rhythmic differences.

#### Motor Learning task

Beginning with their non-dominant hand, children performed three, 30-second trials of a 4-finger sequence task on the number keys of an alpha-numeric computer keyboard [33]. In order to avoid potential confusion for musicians who associate numbers with specific fingers, colored stickers were placed over the numbers on the four target keys, and a matching set of stickers was placed on the child's fingers to correspond with the pattern. Each target pattern was represented by a series of colored stickers on a card that served as a visual reminder during the task. Children were asked to repeat the correct sequence of key presses as many times as possible in 30 seconds. The task was performed three times with each hand, with a 30-second rest period between trials. Full credit (a score of 1) was given for each correct, 5-key press sequence; partial credit was given for sequences with four consecutive key presses (score of 0.8) and three consecutive key presses (score of 0.6). Scores from the three trials were averaged to obtain a mean score for each hand.

#### Block Design

Children received the Block Design subtest of the WISC-III [36] (age-scaled). Rauscher, Shaw, Levine, Wright, Dennis, et al. [37] argued that this is a spatial recognition task since the physical model (picture) of the design to be copied remains present during the task. It therefore does not require the formation of a mental image. Schellenberg [29] showed that performance on this task (as well as on all but one of the other WISC subtests) improved after music training.

#### Object Assembly

Children received the Object Assembly subtest of the WISC-III [36] (age-scaled). This task requires both the formation of a mental image and the manipulation of that image within a limited time period [37]. Performance on this task has been shown to be improved by music training [29], [37].

#### Raven's Progressive Matrices

Three levels of the Raven's Progressive Matrices (RPM) were administered in the following order: Colored Progressive Matrices, Standard Progressive Matrices and Advanced Progressive Matrices-Set I [38]–[40]. The RPM is considered to be a nonverbal reasoning task with visual-spatial elements [13]. Alternatively, one can think of this task as a visual pattern completion task. As mentioned earlier, a meta-analysis by Hetland [13] demonstrated that performance on Raven's Standard Progressive Matrices was not improved after music training.

#### Vocabulary

Children were given the Vocabulary subtest of the WISC-III [36] (age-scaled), which consists of up to 30 words to be defined orally.

#### Auditory Analysis Test

Children received the Auditory Analysis Test [41], a measure of phonemic awareness. Children hear a list of 40 spoken words (e.g., smell) and are asked to repeat the word and then say it again without one of its sounds (e.g., Say “smell”; now say it again, but without the “m”).

#### KeyMath–Revised

Children received *Keymath-Revised: A Diagnostic Inventory of Essential Mathematics* [42], which is a comprehensive assessment of children's understanding and application of mathematical concepts and skills. This test is divided into three concept areas (age-scaled): 1) *Basic Concepts*-assesses foundation knowledge (numeration, rational numbers and geometry); 2) *Operations*-assesses computational skills (addition, subtraction, multiplication, division, and mental computation-which covers all four operations); and 3) *Applications*-assesses the ability to apply mathematical knowledge and skills (measurement, time and money, estimation, data interpretation, and problem solving). Because this test was introduced in our battery later, only about two thirds of our sample are included in the math analyses.

## Results

### Preliminary Analyses

A first set of preliminary analyses examined whether or not the type of instrumental instruction received (Suzuki vs. traditional) affected the outcomes measured. One-way ANOVAs (with the type of instruction as the between-subjects factor) showed that the two Instrumental subgroups did not differ in age, gender, SES or duration of training (all *p*>.1). Two MANOVAs were conducted in order to assess whether any between-groups differences existed. Mathematical outcomes had to be analyzed in a separate MANOVA because 16 out of the 41 children had not taken the Keymath-R test. The main MANOVA (including as dependent measures scores on Block Design, Vocabulary, Object Assembly, Raven's Colored, Standard and Advanced Progressive Matrices, Gordon's tonal and rhythm subtests, as well as on the Melodic and Rhythmic Discrimination task) revealed no overall between-groups difference, Wilks' lambda = .72, *F* (13, 27) = .8, *p* = .66. Missing values had been replaced by the series' mean (for 8.26% of all values). The second MANOVA, which included the math results for 25 children (9 traditional, 16 Suzuki) also did not yield an overall difference, Wilks' Lambda = .92, *F* (3, 21) = .62, *p* = .61. The two Instrumental subgroups were therefore combined in all further analyses.

Preliminary ANOVAs comparing the Instrumental and Control groups showed no difference between groups in SES (*p*>.1); Control SES mean = 4.64 points, *SD* = 0.74, Instrumental SES mean = 4.78 points, *SD* = 0.78. A chi square analysis showed that the distribution of males and females across groups did not differ (*p*>.1). Groups however differed in age, *F* (1, 57) = 5.67, *p* = .021: the Instrumental group (*M* = 10.10 years old, *SD* = 0.76) was somewhat older than the Control group (*M* = 9.63 years old, *SD* = 0.57). Age was therefore covaried in all analyses, even for outcomes with age-scaled scores.

### Between-Group Analyses

A MANCOVA (covarying age) was conducted to test for between-groups differences on all outcomes (except math since our sample was smaller for this test, as explained earlier). Missing values were replaced by the series' means (for 5.87% of all values). There was an overall significant effect of group, Wilks' Lambda = .54, *F* (13, 44) = 2.94, *p*<.01. Subsequent univariate tests showed that groups differed on 7 out of the 13 outcomes. The Instrumental group outperformed the Control group in four of the closely associated domains (left and right hand Motor Learning, Gordon's IMMA tonal subtest, the Melodic Discrimination task) and in three distantly associated domains (Vocabulary, Raven's Standard and Advanced Progressive Matrices) (all *p*<.05). Groups did not differ in four other distant outcomes (Block Design, Object Assembly, Raven's Colored Matrices, and Auditory Analysis), nor did they differ in the two closely related outcomes of rhythm discrimination. Estimated marginal means (covarying age), standard errors, *F* and *p* statistics for all outcomes are listed in Table 1.

**Table 1:** Instrumental vs. Control Group Results as Shown by Univariate Post-Test Results After MANCOVAs.

Outcomes	Control		Instrumental		Results	
	*M*	*SE*	*M*	*SE*	*F*	*p*
Left Hand Motor Learning	7.05	.93	10.27	.60	8.17	<.01
Right Hand Motor Learning	7.48	.10	11.93	.65	13.57	<.01
IMMA Tonal	33.76	.66	37.14	.43	17.732	<.01
IMMA Rhythm	33.24	.71	33.78	.46	.391	.53
Melodic Discrimination	71.29	2.82	82.16	1.83	10.04	<.01
Rhythmic Discrimination	64.42	3.60	71.69	2.34	2.75	.10
Vocabulary	13.47	.61	15.50	.40	7.39	<.01
Auditory Analysis	31.01	1.10	32.99	.71	2.21	.14
Block Design	13.84	.76	14.22	.49	.168	.68
Object Assembly	10.87	.69	11.74	.45	1.07	.31
Raven's Colored PM	33.06	.53	34.13	.35	2.72	.11
Raven's Standard PM	22.64	1.04	25.15	.68	3.97	.05
Raven's Advanced PM	8.44	.39	9.45	.26	4.50	.04
KeyMath-Basic Concepts	123.16	2.47	127.62	1.94	1.89	.18
KeyMath-Operations	115.73	3.76	123.69	2.96	2.59	.12
KeyMath-Applications	119.17	2.22	122.45	1.75	1.26	.27

A second MANCOVA (covarying age) was carried out in order to compare groups on mathematical outcomes (the Basic Concepts, Operations and Applications areas of the Keymath-R test). A separate analysis had to be conducted because only 41 out of the 59 children received this test (16 Controls and 25 Instrumentals). The MANCOVA did not reveal an overall significant effect, Wilks' Lambda = .92, *F* (3, 36) = 1.06, *p* = .38. None of the subsequent univariate tests were significant (all *p*>.1). Estimated marginal means (covarying age), standard errors, *F* and *p* statistics for all mathematical outcomes are also listed in Table 1.

In the above analyses, we did not attempt to equate the Instrumental and Control groups for either verbal or non-verbal IQ since these outcomes may well be effects of music training. However, to determine whether findings could be explained by pre-existing differences in either verbal or non-verbal IQ, we repeated the above analyses, once adding Vocabulary as a covariate, and once adding Raven's Progressive Matrices as a covariate (and covarying age both times, as in the previous analyses). When Vocabulary was added as a covariate, the Instrumental group was no longer superior on any of the Raven's Progressive Matrices (but remained superior on the motor and the auditory discrimination tests). However, when Raven's Colored, Standard, or Advanced Matrices scores were added as covariates (one at a time), the Instrumental group remained superior on the Vocabulary subtest of the WISC (*p*≤.05) as well as on the motor and auditory discrimination tests. Thus, when non-verbal intelligence was controlled, associations remained between music training and verbal, motor as well as auditory outcomes. While the superiority of musically trained children in nonverbal reasoning could potentially be explained by pre-existing differences in verbal ability, the reverse was not true: musically trained children are superior in verbal ability to those without training, even after controlling for their superiority in nonverbal reasoning.

### Predictors of Practice Intensity at Time of Testing

We investigated the relationship, within the Instrumental group, between weekly practice intensity at the time of testing and other independent variables. Practice intensity (in minutes per week) was significantly correlated with training duration (*r* ^2^ = .17, *p* = .03), but not with age, SES or gender (all *p*>.1). Thus, children who persist in studying an instrument for more years are also more capable of, and/or more willing to practice for a longer daily period.

### Effect of Training Duration on Outcomes

A series of multiple regressions were performed in order to determine the effects of training duration on outcomes, controlling for age. Those in the Control group were entered as having zero weeks of training.

**Figure 1:**
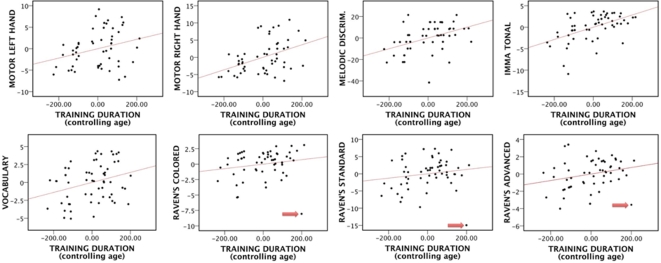
Significant partial correlations (controlling age) between training duration (in weeks) and motor learning left/right hand, Melodic Discrimination, the IMMA tonal subtest, Vocabulary and Raven's Progressive Matrices.

Controlling for age, training duration predicted four near outcomes: Motor learning on both left (partial *r* ^2^ = .08, *p* = .04) and right (partial *r* ^2^ = .19, *p*<.01) hands, Gordon's IMMA Tonal subtest (partial *r* ^2^ = .26, *p*<.01) and the Melodic Discrimination Task (partial *r* ^2^ = .18, *p*<.01). Training duration also predicted some of the distant outcomes: Vocabulary (partial *r* ^2^ = .09, *p* = .02) and Raven's Advanced PM, although only at a near-significant level (partial *r* ^2^ = .06, *p* = .06). Figure 1 illustrates significant partial correlations. Our lack of significant findings with the Raven's Progressive Matrices was surprising, since we had found between-groups effects. Upon closer inspection of the regression plots, we observed that one outlier (marked with a red arrow in Figure 1) could be responsible for this lack of effect. This particular subject scored below *M*-2*SD* on all three subtests of Raven's PM. Indeed, after removing the outlier, training duration significantly predicted Raven's Colored (partial *r* ^2^ = .13, *p*<.01), Standard (partial *r* ^2^ = .10, *p* = .02), and Advanced (partial *r* ^2^ = .12, *p* = .01) Progressive Matrices.

**Figure 2:**
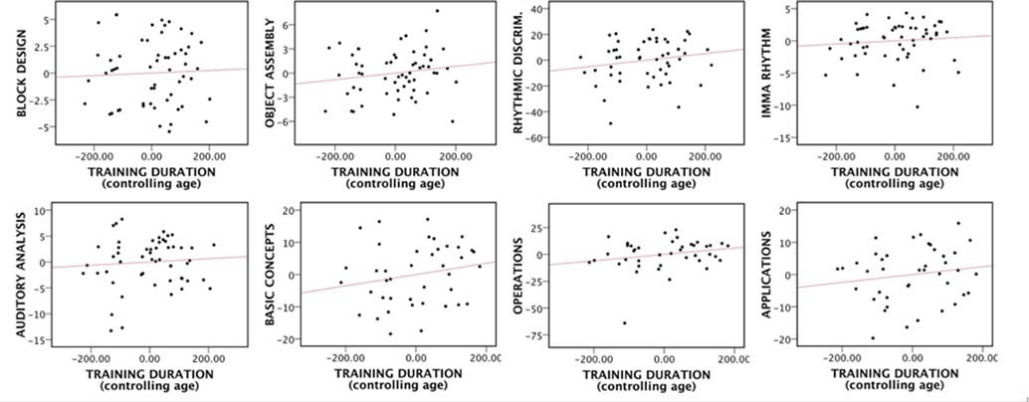
Nonsignificant partial correlations (controlling age) between training duration (in weeks) and Block Design, Object Assembly, Rhythmic Discrimination, the IMMA rhythm subtest, Auditory Analysis, and the Keymath-R Basic Concepts, Operations and Applications.

Duration of training did not predict any other outcomes (all *p*>.1). Figure 2 illustrates non-significant findings. This was not surprising since we had not found between-group differences on these measures.

We did not include practice intensity as an independent variable in the above regression analyses for two reasons: (1) practice intensity was only reported at the time of testing and therefore did not accurately reflect the amount of practice achieved over the years; (2) training duration and practice intensity were significantly correlated with each other and thus entering both factors at the same time would be redundant and reduce power.

## Discussion

The results of the present study showed that children who had received instrumental music training for three years or more outperformed their control counterparts in areas closely related to music: fine motor skills (both hands) and discrimination between melodies (both on the Gordon's IMMA and the Melodic Discrimination Task). Strengthening these results is the finding that duration of music training predicted these results as well. These results are consistent with previous reports in the literature that music training is associated with enhanced fine motor skills in children [10], [11] and in adults [12], and with studies showing that musically trained children have superior melodic/tonal and rhythmic discrimination abilities [8], [9].

The results also showed that instrumental children outperformed their control counterparts in verbal ability (Vocabulary) and in non-verbal reasoning (both Raven's Standard and Advanced PM). Strengthening these results is the finding that duration of music training predicted performance on the Vocabulary test and on Raven's Advanced Progressive Matrices.

Contrary to some previous research, the Instrumental group did not outperform the Control group on phonemic awareness (as measured by the Auditory Analysis Test) or spatial skills (as measured by the WISC-III Block Design and Object Assembly subtests), nor were duration of training effects found for these outcomes. While Hetland [13] previously suggested a significant effect of music training on spatial tasks, it is important to note that the fifteen studies included in her meta-analysis were all shorter in duration (from six weeks to two years) than the average 4.6 years of musical training received by our participants. Furthermore, Costa-Giomi [43] found that children receiving piano lessons improved more than controls in visual-spatial skills over the first two years of instruction, but the controls caught up with the experimental group by the end of the third year. It is therefore possible that instrumental music training may accelerate the natural development of spatial abilities rather than confer a permanent advantage to musicians. Costa-Giomi [43] also suggested that her lack of an effect in the third year of follow-up might be explained by hormonal changes. As her subjects entered adolescence, the relationship between music training and spatial performance may have been altered. Buttressing Costa-Giomi's explanation is Hassler's [44] finding that the onset of puberty reduces the difference in spatial abilities between musicians and nonmusicians. The results of our study cannot be explained by the hormonal changes that occur during puberty, however, as our children were only about ten years of age. In addition, the significant (although modest) relationship between music training and mathematical abilities reported by Vaughn [3] was not supported by this study.

Three different types of explanations, which are detailed below, could account for our findings. The superiority of the Instrumental group may be due to: (1) domain-specific transfer effects (instrumental music training may causally enhance selected cognitive abilities) (2) a domain-general transfer effect (instrumental music training may enhance general IQ and lead to improvements in all cognitive domains); (3) non-causal associations mediated by third variables which were not accounted for in this study.

The first two explanations, which a correlational study can only suggest but not demonstrate, propose that transfer effects may have occurred between music training and distantly related domains. This explanation is supported by the fact that training duration predicted performance in these outcomes. The association between music training and vocabulary is consistent with past research suggesting that instrumental music training enhances verbal memory [20], [21], [25], [26], phonological awareness [23], [24], and reading skills [22]. The effects found with Raven's Progressive Matrices were surprising, as a meta-analysis by Hetland [13] reported that music training did not affect performance in this task. However, as pointed out by Hetland, the meta-analysis only included five effect sizes derived from a total of three published studies. In addition, participants in these three studies had received on average less (from 7 months to three years) than the average 4.6 years of music training received by children in our study. More research is therefore needed in order to ascertain the relationship between music training and performance on Raven's Progressive Matrices.

### The Domain-Specific Hypothesis

The first explanation suggested by our results is that transfer effects may have occurred between music training and a selection of related domains. These transfers can be explained by the fact that some aspects of music are shared with other activities. Learning to decode written music notation may, for instance, increase reading ability. Learning to categorize sounds may enhance phonological awareness in nonmusical settings. The honing of visual pattern recognition and pattern matching skills resulting from instrumental practice and notation reading may explain our surprising results on the Raven's Progressive Matrices, since many of the items on this test can be solved using a visual pattern recognition/matching strategy. In the present study, the domain-specific hypothesis was supported by the fact that verbal ability was superior in the Instrumental group even when nonverbal reasoning skills were controlled.

### The Domain-General Hypothesis

A second hypothesis can explain our findings and clarify how transfer effects take place. As suggested by Schellenberg [29], music training may enhance not just one set of skills, but general intellectual ability. Children who take up music should therefore experience improvements in all domains. Although the Instrumental group in our study did not outperform the Control group on all outcomes, we cannot discard the general IQ explanation given that musicians had higher means on all outcomes (See Table 1). Further research is needed to clarify whether transfer effects are domain-general or domain-specific.

### Non-Causal Explanations

The correlational design of this study does not allow us to rule out a variety of non-causal explanations for the associations found. First, family dynamics may account for the findings. Children whose parents enroll them in music lessons may be more engaged with their children's education, and provide more enriched environments than do parents who do not enroll their children in music lessons. Children who practice their instruments more than others may do so because of parental expectations and insistence. These same parents may insist that their children work hard in school, do their homework, and read. Miller and Orsmond [46] for example found that children taking up music instruction were more likely to have additional extra-curricular activities as well as to benefit from higher levels of parental involvement than a matched control group.

Second, superior motivational skills in instrumental children may account for our findings. Our study only included children who had persisted with music lessons for at least three years. We did not include those who had begun lessons but dropped them early on. Thus the children in the Instrumental group may have been more persistent and motivated than average. Children with superior motivational skills and ability to persist on difficult tasks may not only stick with music lessons but may also practice more than is typical of children taking lessons. In addition, such children may work harder at school and read more, thereby learning more and resulting in heightened performance on the kinds of cognitive tests administered in this study.

The results of our study confirm previous research showing that children who take instrumental music lessons are ahead on a number of cognitive abilities. However, the correlational design of this study does not allow us to determine definitively whether music causally enhanced verbal and nonverbal reasoning skills, or whether other variables were responsible for the effects found. The causal explanation was supported by the fact that duration of training linearly predicted cognitive outcomes.

The results of this study, and the issues they raised, will guide our expectations for an ongoing quasi-experimental longitudinal study currently underway in our laboratory. At the final timepoint of this longitudinal study, the children participating will have received about the same amount of music training as the children included in the present study. At baseline, children in the Instrumental group did not differ from those in the Control group on any outcomes [33]. This longitudinal study will allow us to determine whether the associations between music training and extra-musical outcomes found here are causal or non-causal in nature. This study will also shed light on whether instrumental music training has domain-specific or domain-general general effects.
